# Development and validation of a clinical prediction tool for non-receipt of updated COVID-19 vaccines

**DOI:** 10.1016/j.vaccine.2025.127074

**Published:** 2025-04-11

**Authors:** Katia J. Bruxvoort, Lina S. Sy, Richard Contreras, Bruno Lewin, Vennis Hong, Lei Qian, Kimberly J. Holmquist, Bing Han, Stanley Xu

**Affiliations:** a Department of Epidemiology, University of Alabama at Birmingham, Birmingham, AL 35233, United States; b Department of Research & Evaluation, Kaiser Permanente Southern California, 100 S. Los Robles Ave, 5th Floor, Pasadena, CA 91101, United States; c Department of Health Systems Science, Kaiser Permanente Bernard J. Tyson School of Medicine, Pasadena, CA 91101, United States

**Keywords:** COVID-19 vaccine, Uptake, Prediction model

## Abstract

Vaccination with updated COVID-19 vaccines is important to maintain protection against circulating SARS-CoV-2 variants. We developed and validated a prediction model for non-receipt of updated COVID-19 vaccines at Kaiser Permanente Southern California. Of 3,287,287 adults, 20.2 % received 2023–2024 COVID-19 vaccine. We assessed the association of 15 variables available in electronic health records with non-receipt of 2023–2024 updated COVID-19 vaccine. The cohort was split into development and validation samples. Performance of the 15-variable model in the development sample was moderate, with a scaled Brier score of 35.6 %, R^2^ of 29.3 %, C-statistic of 0.882, discrimination slope of 0.354, and calibration slope of 1; performance was similar for the validation sample and simplified 6-variable and 2-variable models. The strongest predictors were prior non-receipt of bivalent COVID-19 vaccine or influenza vaccine. These results suggest that a simple model using variables available to healthcare providers can be optimized to guide intervention strategies for updated COVID-19 vaccines.

## 1. Introduction

The United States (U.S.) Centers for Disease Control and Prevention (CDC) recommends staying up-to date with respiratory virus vaccinations, including COVID-19 vaccination. [1] Following recommendations for receipt of mRNA original monovalent vaccines in 2020–2021 [2–4] and bivalent original and Omicron BA.4/BA.5 COVID-19 vaccine in 2022, [5] updated vaccines were recommended in 2023–2024 targeting XBB.1.5 [6] and in 2024–2025 targeting KP.2. [7] Vaccination with updated COVID-19 vaccines is important to account for waning immunity from previous COVID-19 vaccinations and prior SARS-CoV-2 infection and to provide broader protection against emerging SARS-CoV-2 variants. [8,9]

Although COVID-19 vaccines are safe and effective, preventing an estimated 3.2 million deaths and 18 million hospitalizations in the U.S. from 2021 to 2022, [10] uptake has been challenging. [11,12] Prior studies identified disparities in COVID-19 vaccine uptake, with people from some racial/ethnic minority groups and those with high social vulnerability less likely to get vaccinated. [12–14] Among Kaiser Permanente Southern California (KPSC) members who received the original COVID-19 vaccine series, uptake of bivalent COVID-19 vaccine was 19.5 % among youth and 30.7 % among adults. [15] CDC estimates that only 22.5 % of adults received the 2023–2024 vaccine. [16] Reasons for declining COVID-19 vaccine uptake include concerns about safety and effectiveness, often fueled by misinformation, and systemic issues such as inequitable access to healthcare and eroding trust in healthcare authorities. [17]

As the SARS-CoV-2 virus continues to evolve, causing large winter and smaller summer surges of COVID-19, updated COVID-19 vaccines may be recommended seasonally for the foreseeable future. To address challenges with vaccine uptake, a clinically-relevant prediction tool to identify individuals who are less likely to get vaccinated could help healthcare providers and administrators develop and target effective strategies for reaching these groups. Thus, we conducted a study to develop and internally validate a prediction model for not receiving updated COVID-19 vaccine during the 2023–2024 respiratory virus season.

## 2. Methods

### 2.1. Study setting

KPSC is an integrated healthcare organization serving over 4.8 million sociodemographically diverse members who are generally representative of the underlying population. [18] KPSC has 15 hospitals with associated medical offices across 9 counties, and members are enrolled through commercial, Medicaid, and Medicare plans. Comprehensive electronic health records (EHR) collect detailed information on care received, including visits, diagnoses, vaccinations, laboratory tests and results, procedures, and medications. Members are incentivized to receive care within the KPSC system, but outside care is captured through claims. External COVID-19 vaccinations from all healthcare providers and pharmacies are imported from the California Immunization Registry (CAIR) and automatically incorporated into KPSC immunization records. The study was approved the KPSC Institutional Review Board (IRB #13270).

### 2.2. Study design

The study included adults aged ≥18 years with ≥1 year of KPSC membership prior to the index date, defined as 9/12/2023 – the date of the Advisory Committee on Immunization Practices [ACIP] recommendation for the 2023–2024 COVID-19 vaccine targeting XBB.1.5 (hereafter, updated COVID-19 vaccine). Individuals were excluded if EHR data indicated that they received updated COVID-19 vaccine prior to 9/12/2023, as these may have reflected errors in entry of vaccination records. The primary outcome was receipt of updated COVID-19 vaccine from the index date until 4/30/2024.

### 2.3. Variables

We considered a range of potential predictor variables for not receiving updated COVID-19 vaccine, based on our prior work. [15] Variables collected at index date included age group (18–24, 25–44, 45–64, 65–84, and ≥ 85 years), sex, race/ethnicity (Asian/Pacific Islander, Black, Hispanic, White, and Multiple/Other/Unknown), Medicaid insurance, and neighborhood deprivation index (NDI) quintile (Q1 for least deprived, Q5 for most deprived). Variables collected in the year prior to index date included Charlson Comorbidity Index (CCI), [19] immunocompromise (defined as leukemia, lymphoma, congenital immunodeficiencies, asplenia/hyposplenia, HIV/AIDS, solid organ transplant, or receipt of immunosuppressive medications), [20] and healthcare utilization (number of inpatient, emergency department [ED], outpatient, and virtual visits). We also collected data on prior COVID-19 (i.e., SARS-CoV-2 positive test or COVID-19 diagnosis code), receipt of seasonal influenza vaccine during 8/1/2022 to 4/30/2023, receipt of bivalent COVID-19 vaccine prior to index date, and number of prior COVID-19 vaccine doses.

### 2.4. Development of prediction model

We described the distribution of characteristics among individuals in the cohort overall and by receipt of updated COVID-19 vaccine during the study period. We then randomly split the study cohort 50/50 into development and validation datasets. Using the development sample, we assessed factors associated with not receiving updated COVID-19 vaccine, using 15-variable and 6-variable logistic regression models. The 15-variable model included all variables described above. The 6-variable model included age, race/ethnicity, Medicaid insurance, number of prior year inpatient visits, prior receipt of 2022–2023 seasonal influenza vaccination, and prior receipt of bivalent COVID-19 vaccine; these 6 variables were selected based on significance and strength of association with not receiving updated COVID-19 vaccine in the 15-variable model, and because they are readily available in EHR data.

### 2.5. Model performance and internal validity

We then assessed the performance of the 15-variable and 6-variable models in the development and validation datasets. We examined overall model performance using the Brier score and R^2^-statistics, discrimination using the concordance (or C) statistic and discrimination slope, and calibration using the calibration slope. [21] We used a range of cut-offs between 0.70 and 0.90 for the proportion of individuals predicted not to receive updated COVID-19 vaccine to examine the sensitivity and specificity of the predictive models. These cut-offs were selected based on observed rates of non-vaccination in the study population, allowing flexibility to prioritize populations for intervention.

## 3. Results

The study cohort included 3,287,287 adults (Supplementary Fig. 1). Of these, 665,341 (20.2 %) received updated COVID-19 vaccine during the study period. A greater proportion of individuals who received updated COVID-19 vaccine than those who did not receive updated COVID-19 vaccine were older (e.g., age 65–84 years, 41.2 % vs 14.9 %), White (38.3 % vs 28.3 %), had higher socioeconomic status (e.g., Q1, 29.7 % vs 20.9 %), had more comorbidities (e.g., CCI ≥3, 11.7 % vs 4.2 %), were immunocompromised (4.5 % vs. 1.9 %), had more prior year inpatient, ED, outpatient, and virtual visits, had received influenza vaccine in the 2022–2023 season (86.7 % vs 37.8 %), and had received COVID-19 bivalent vaccine (74.4 % vs 15.2 %) (Table 1).

In the 15-variable model (Table 2), factors associated with not receiving updated COVID-19 vaccine included younger age (e.g., age 18–24 vs ≥85 years, adjusted odds ratio [aOR] 2.25, 95 % CI: 2.18–2.33), female vs male sex (aOR 1.07, 95 % CI: 1.06–1.08), race/ethnicity (e.g., Hispanic vs White, aOR 1.23, 95 % CI: 1.22–1.25; Black vs White, aOR 1.13, 1.11–1.15), Medicaid insurance (aOR 1.04, 95 % CI: 1.02–1.06), higher NDI (e.g., Q5 vs Q1, aOR 1.28, 95 % CI: 1.26–1.30), more prior year inpatient and ED visits (e.g., ≥1 vs 0 inpatient visits, aOR 1.21, 1.19–1.24; ≥1 vs 0 ED visits, aOR 1.10, 1.09–1.12), lower CCI (e.g., 0 vs ≥3, aOR 1.11, 95 % CI: 1.08–1.13), not being immunocompromised (aOR 1.07, 95 % CI: 1.05–1.10), prior COVID-19 (aOR 1.03, 95 % CI: 1.01–1.04), not receiving 2022–2023 influenza vaccine (aOR 2.79, 95 % CI: 2.76–2.83), not receiving bivalent COVID-19 vaccine (aOR 5.32, 95 % CI: 5.27–5.38), and prior receipt of fewer COVID-19 vaccine doses (e.g. 0 vs ≥3, aOR 16.80, 15.93–17.72).

In the 6-variable model (Table 2), factors associated with not receiving updated COVID-19 vaccine included younger age (e.g., age 18–24 vs ≥85 years, aOR 2.74, 95 % CI: 2.66–2.83), race/ethnicity (e.g., Hispanic vs White, aOR 1.28, 95 % CI: 1.27–1.30; Black vs White, aOR 1.15, 95 % CI: 1.13–1.17), Medicaid insurance (aOR 1.11, 95 % CI: 1.08–1.13), ≥1 vs 0 prior year inpatient visits (aOR 1.17, 95 % CI: 1.14–1.19), not receiving 2022–2023 influenza vaccine (aOR 3.93, 95 % CI: 3.88–3.98), and not receiving bivalent COVID-19 vaccine (aOR 8.19, 95 % CI: 8.11–8.27).

Overall, the 15-variable model performed well (Table 3). For the development sample, the scaled Brier score (range 0–100 % with lower values indicating better model performance) was 35.6 %, suggesting that the model’s predicted probabilities were moderately close to the observed outcomes. The R^2^ statistic quantifying the proportion of variability in the outcome explained by the model was 29.3 %, indicating moderate explanatory power but also highlighting that a substantial portion of the variability remained unexplained. Model discrimination between individuals who did not receive the updated COVID-19 vaccine compared to those who did was also good, with a C-statistic of 0.882 (range 0–1, with 1 being perfect concordance). The discrimination slope was 0.354, suggesting that the model could distinguish between the two groups, but there was still potential for improving the model’s discriminatory power. The calibration slope was 1, showing high agreement between predicted and observed outcomes. Performance metrics were similar for the validation sample and the 6-variable model (Table 3).

**Fig. 1.**
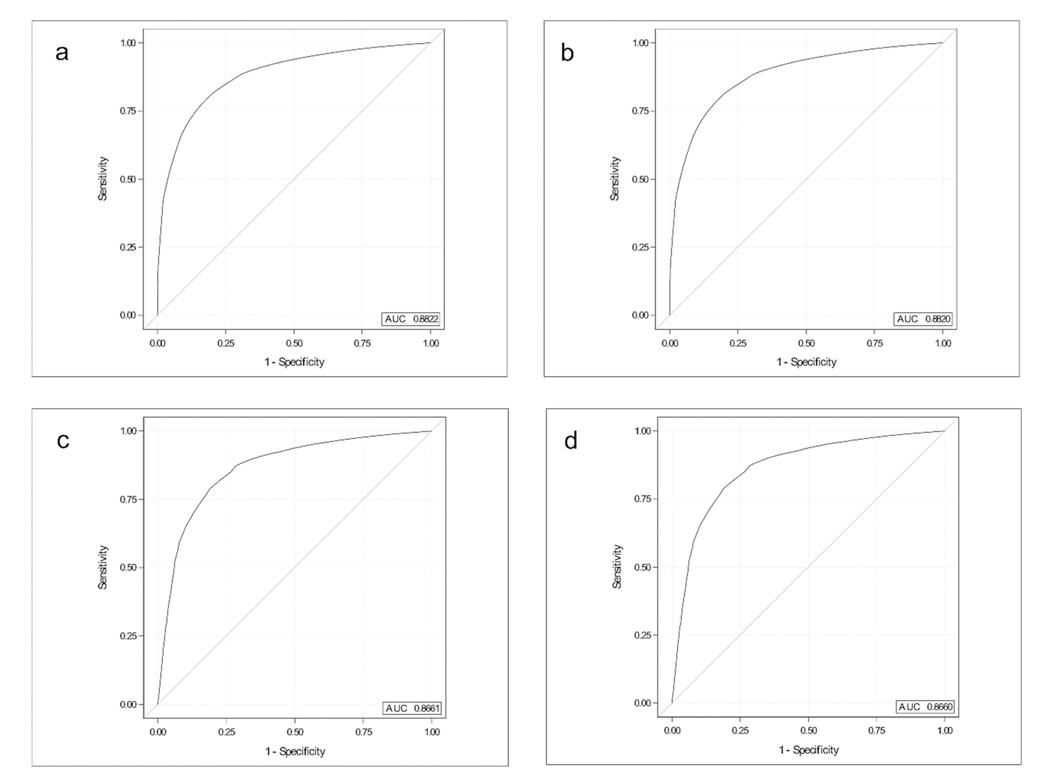
Receiver operating characteristic (ROC) curves for (a) 15-variable model development sample, (b) 15-variable model validation sample, (c) 6-variable model development sample, (d) 6-variable model validation sample.

The sensitivity and specificity of the predictive models are presented in Fig. 1. The area under the curve (AUC) was 0.882 for both the 15-variable model development and validation samples and 0.866 for the 6-variable model development and validation samples.

Because not receiving 2022–2023 influenza vaccine and not receiving bivalent COVID-19 vaccine were the strongest predictors of not receiving updated COVID-19 vaccine, we conducted a post-hoc analysis with only these 2 variables. The associations between not receiving 2022–2023 influenza vaccine and not receiving bivalent COVID-19 vaccine with not receiving updated COVID-19 vaccine were stronger (aOR 4.65, 95 % CI: 4.60–4.71 and aOR 9.27, 95 % CI: 9.18–9.36, respectively) than in the 15-variable and 6-variable models (Supplementary Table 1). The performance of the 2-variable model was similar, but slightly lower, compared to the 15-variable and 6-variable models, with a Brier score of 32.1 %, C-statistic of 0.848, discrimination slope of 0.323, and calibration slope of 1 (Supplementary Table 2).

## 4. Discussion

In this study among a large cohort in southern California, we developed and internally validated a clinical prediction model for non-receipt of updated COVID-19 vaccine, using common EHR variables. We found that our predictive models performed well at identifying individuals who did not receive updated COVID-19 vaccine. While the 15-variable model had slightly better metrics, the 6-variable model performed nearly as well and offers greater practicality for implementation. Even the 2-variable model, which includes only prior influenza and bivalent COVID-19 vaccination, retained strong predictive ability, underscoring the importance of these two factors in forecasting updated COVID-19 vaccine uptake. Our results indicate that simple models accounting for receipt of prior COVID-19 vaccine and seasonal influenza vaccine, demographic characteristics, and inpatient visits could be useful for healthcare providers and administrators to predict uptake of updated COVID-19 vaccines and to guide development of interventions to reach priority populations.

Few other studies have developed models to predict COVID-19 vaccine uptake, particularly for updated COVID-19 vaccines in learning healthcare systems. One study used machine learning approaches (XGBoost) and sociodemographic data to predict primary series COVID-19 vaccine uptake across U.S. counties with 62 % accuracy. [22] Another study used gradient boosting and survey data from U.S. adults to accurately classify vaccine-accepting individuals (97 % accuracy) and vaccine-hesitant individuals (72 % accuracy); this study identified that trust in and knowledge of vaccines was the primary driver of vaccination choice. [23] Another study used a balanced random forest approach and survey data including judgement, demographic, and COVID-19 precaution variables to predict primary series COVID-19 vaccine uptake with moderate to high recall, specificity, accuracy, and precision; this study found that reward and aversion judgement variables contributed almost two-thirds of the prediction. [24]

In contrast to these studies, our study utilized EHR variables available to healthcare providers and administrators to identify individuals who might not receive updated COVID-19 vaccine. In addition to factors such as younger age, Hispanic or Black race/ethnicity, Medicaid insurance, and prior inpatient visits, we found that not receiving prior season influenza vaccine and not receiving prior bivalent COVID-19 vaccine were the strongest predictors. Similarly, a study using data from general practices in England found that prior season influenza vaccination and pneumococcal vaccination status were the best predictors of seasonal influenza vaccine uptake. [25] These findings support prioritizing interventions for individuals who have not received prior COVID-19 or seasonal influenza vaccines. Potential interventions could include offering COVID-19 vaccines at inpatient and ED visits, individualized education about updated vaccines at healthcare visits and through outreach, and leveraging trusted community messengers.

Our study has several limitations. Misclassification is possible for the updated COVID-19 vaccine outcome, as well as the bivalent COVID-19 vaccine and 2022–2023 influenza vaccine predictors. However, this is unlikely as KPSC has comprehensive EHR, and external vaccinations from healthcare providers and pharmacies are integrated into the EHR from CAIR or with supporting documentation. Since our intent was to create a clinical prediction tool, we did not include knowledge, trust, judgement, and behavioral variables that are not readily available in EHR. In addition, we focused on the 2023–2024 COVID-19 vaccine. Future work could improve the prediction model by incorporating additional variables and validating the model using vaccination data from the 2024–2025 season and beyond.

In conclusion, we developed and internally validated 15-variable, 6-variable, and 2-variable clinical prediction models using commonly available EHR variables that performed well at identifying individuals who did not receive updated COVID-19 vaccine. Prior receipt of bivalent COVID-19 vaccine and 2022–2023 influenza vaccines were the most important predictors, in addition to sociodemographic characteristics and prior year inpatient visits. Future work is warranted to optimize the model for updated vaccines and to guide interventions to increase vaccine uptake.

## Supplementary Material

MMC1

## Figures and Tables

**Table 1:** Sociodemographic and clinical characteristics of study cohort.

	Received updated COVID-19 vaccine	Did not receive updated COVID-19 vaccine	Total

	*N* = 665,341	*N* = 2,621,946	*N* = 3,287,287
	n (%)	n (%)	n (%)
**Age at index date**^1^ **(years)**			
18–24	27,912 (4.2)	336,071 (12.8)	363,983 (11.1)
25–44	120,418 (18.1)	981,847 (37.4)	1,102,265 (33.5)
45–64	211,784 (31.8)	868,982 (33.1)	1,080,766 (32.9)
65–84	274,247 (41.2)	391,933 (14.9)	666,180 (20.3)
≥85	30,980 (4.7)	43,113 (1.6)	74,093 (2.3)
**Sex**			
Female	364,182 (54.7)	1,369,731 (52.2)	1,733,913 (52.7)
Male	301,159 (45.3)	1,252,215 (47.8)	1,553,374 (47.3)
**Race/ethnicity**			
Asian/Pacific Islander	113,672 (17.1)	286,350 (10.9)	400,022 (12.2)
Black	50,315 (7.6)	205,707 (7.8)	256,022 (7.8)
Hispanic	211,363 (31.8)	1,155,633 (44.1)	1,366,996 (41.6)
White	254,827 (38.3)	743,303 (28.3)	998,130 (30.4)
Multiple/Other/Unknown	35,164 (5.3)	230,953 (8.8)	266,117 (8.1)
**Medicaid insurance**	40,422 (6.1)	236,855 (9.0)	277,277 (8.4)
**NDI quintile**			
Q1	197,306 (29.7)	549,081 (20.9)	746,387 (22.7)
Q2	157,238 (23.6)	555,849 (21.2)	713,087 (21.7)
Q3	125,647 (18.9)	536,599 (20.5)	662,246 (20.1)
Q4	102,248 (15.4)	504,414 (19.2)	606,662 (18.5)
Q5	82,257 (12.4)	471,416 (18.0)	553,673 (16.8)
Missing	645 (0.1)	4587 (0.2)	5232 (0.2)
**Charlson Comorbidity Index**			
0	339,118 (51.0)	1,940,093 (74.0)	2,279,211 (69.3)
1	164,595 (24.7)	436,712 (16.7)	601,307 (18.3)
2	83,901 (12.6)	135,358 (5.2)	219,259 (6.7)
≥3	77,727 (11.7)	109,783 (4.2)	187,510 (5.7)
**Immunocompromised**	29,774 (4.5)	50,645 (1.9)	80,419 (2.4)
**Number of inpatient encounters in year prior to index date** ^1^			
0	627,841 (94.4)	2,505,934 (95.6)	3,133,775 (95.3)
≥1	37,500 (5.6)	116,012 (4.4)	153,512 (4.7)
**Number of ED encounters in year prior to index date** ^1^			
0	546,035 (82.1)	2,213,448 (84.4)	2,759,483 (83.9)
≥1	119,306 (17.9)	408,498 (15.6)	527,804 (16.1)
**Number of outpatient encounters in 1 year before index date** ^1^			
0	32,436 (4.9)	533,959 (20.4)	566,395 (17.2)
1	41,445 (6.2)	324,773 (12.4)	366,218 (11.1)
2–4	141,874 (21.3)	714,757 (27.3)	856,631 (26.1)
5–9	194,812 (29.3)	570,733 (21.8)	765,545 (23.3)
≥10	254,774 (38.3)	477,724 (18.2)	732,498 (22.3)
**Number of virtual encounters in 1 year before index date** ^1^			
0	230,260 (34.6)	1,269,953 (48.4)	1,500,213 (45.6)
1	131,799 (19.8)	482,601 (18.4)	614,400 (18.7)
2–4	173,450 (26.1)	522,073 (19.9)	695,523 (21.2)
5–9	75,921 (11.4)	204,477 (7.8)	280,398 (8.5)
≥10	53,911 (8.1)	142,842 (5.4)	196,753 (6.0)
**COVID-19 prior to index date** ^1^	231,365 (34.8)	900,052 (34.3)	1,131,417 (34.4)
**Received 2022–2023 influenza vaccine**	576,908 (86.7)	991,970 (37.8)	1,568,878 (47.7)
**Received bivalent COVID-19 vaccine**	494,945 (74.4)	399,049 (15.2)	893,994 (27.2)
**Number of COVID-19 vaccine doses prior to index date** ^1^			
0	2836 (0.4)	530,122 (20.2)	532,958 (16.2)
1	3247 (0.5)	112,920 (4.3)	116,167 (3.5)
2	24,389 (3.7)	709,192 (27.0)	733,581 (22.3)
≥3	634,869 (95.4)	1,269,712 (48.4)	1,904,581 (57.9)

Abbreviations: ED, emergency department; NDI, neighborhood deprivation index.

1 Index date was defined as 09/12/2023.

**Table 2:** Factors associated with not receiving updated COVID-19 vaccine.

	15-variable model		6-variable model	
	aOR	95 % CI	aOR	95 % CI

**Age at index date**^1^ **(years)**				
18–24	2.25	2.18, 2.33	2.74	2.66, 2.83
25–44	2.02	1.96, 2.08	2.37	2.31, 2.43
45–64	1.55	1.51, 1.60	1.69	1.65, 1.74
65–84	0.95	0.93, 0.98	0.97	0.95, 1.00
≥85	ref		ref	
**Sex**				
Female	1.07	1.06, 1.08		
Male	ref			
**Race/ethnicity**				
Asian/Pacific Islander	1.09	1.07, 1.10	1.01	1.00, 1.03
Black	1.13	1.11, 1.15	1.15	1.13, 1.17
Hispanic	1.23	1.22, 1.25	1.28	1.27, 1.30
White	ref		ref	
Multiple/Other/Unknown	1.18	1.15, 1.20	1.21	1.19, 1.24
**Medicaid insurance**				
Yes	1.04	1.02, 1.06	1.11	1.08, 1.13
No	ref		ref	
**NDI**				
Q1	ref			
Q2	1.09	1.08, 1.11		
Q3	1.15	1.13, 1.17		
Q4	1.21	1.19, 1.23		
Q5	1.28	1.26, 1.30		
Missing	1.48	1.28, 1.71		
**Charlson Comorbidity Index**				
0	1.11	1.08, 1.13		
1	1.00	0.98, 1.02		
2	0.96	0.94, 0.98		
≥3	ref			
**Immunocompromised**				
Yes	ref			
No	1.07	1.05, 1.10		
**Number of inpatient encounters in year prior to index date** ^1^				
0	ref		ref	
≥1	1.21	1.19, 1.24	1.17	1.14, 1.19
**Number of ED encounters in year prior to index date** ^1^				
0	ref			
≥1	1.10	1.09, 1.12		
**Number of outpatient visits in year prior to index date** ^1^				
0	ref			
1	1.03	1.00, 1.05		
2–4	1.04	1.01, 1.06		
5–9	0.99	0.97, 1.01		
≥10	0.91	0.88, 0.93		
**Number of virtual visits in year prior to index date** ^1^				
0	ref			
1	0.99	0.98, 1.00		
2–4	0.98	0.96, 0.99		
5–9	0.94	0.93, 0.96		
≥10	0.81	0.79, 0.83		
**COVID-19 prior to index date**				
Yes	1.03	1.01, 1.04		
No	ref			
**Received 2022–2023 influenza vaccine**				
Yes	ref		ref	
No	2.79	2.76, 2.83	3.93	3.88, 3.98
**Received bivalent COVID-19 vaccine**				
Yes	ref		ref	
No	5.32	5.27, 5.38	8.19	8.11, 8.27
**Number of COVID-19 vaccine doses prior to index date** ^1^				
0	16.80	15.93, 17.72		
1	4.33	4.12, 4.56		
2	3.41	3.34, 3.48		
≥3	ref			

Abbreviations: aOR, adjusted odds ratio; CI, confidence interval; ED, emergency department; NDI, neighborhood deprivation index.

1 Index date was deffned as 09/12/2023.

**Table 3:** Performance of 15-variable and 6-variable models for not receiving updated COVID-19 vaccine.

	15-variable model		6-variable model	
Performance Measure	Development	Validation	Development	Validation

	(*n* = 1,643,644)	(n = 1,643,643)	(n = 1,643,644)	(n = 1,643,643)
**Overall**				
Brier	0.104	0.104	0.106	0.106
Brier scaled	35.6 %	35.3 %	34.3 %	34.3 %
R^2^	29.3 %	29.4 %	27.6 %	27.5 %
**Discrimination**				
C statistic (95 % CI)	0.882 (0.881, 0.883)	0.882 (0.881, 0.883)	0.866 (0.865, 0.867)	0.866 (0.865, 0.867)
Discrimination slope	0.354	0.353	0.343	0.343
**Calibration**				
Calibration slope	1	1	1	1

Abbreviations: CI, confidence interval.

## Data Availability

The data that have been used is confidential.

## Appendix A. Supplementary data

Supplementary data to this article can be found online at https://doi.org/10.1016/j.vaccine.2025.127074.
