# Supplementary material for: Development and validation of a clinical prediction tool for non-receipt of updated COVID-19 vaccines
Source: Vaccine. Author manuscript; Available in PMC 2026 Jul 25. (PMC13401481; doi:10.1016/j.vaccine.2025.127074)
Supplement: MMC1 [file NIHMS2073542-supplement-MMC1.docx]

**Supplementary Material for:**

**Development and validation of a clinical prediction tool for non-receipt of updated COVID-19 vaccines**

Katia J. Bruxvoort, PhD^1,2^, Lina S. Sy, MPH^2^, Richard Contreras, M^2^, Bruno Lewin, MD^2^, Vennis Hong, MPH^2^, Lei Qian, PhD^2^, Kimberly J. Holmquist, MPH^2^, Bing Han, PhD^2^, Stanley Xu, PhD^2,3^

^1^Department of Epidemiology, University of Alabama at Birmingham, Birmingham, AL 35233

^2^Department of Research & Evaluation, Kaiser Permanente Southern California, 100 S. Los Robles Ave, 5th Floor, Pasadena, CA 91101

^3^ Department of Health Systems Science, Kaiser Permanente Bernard J. Tyson School of Medicine, Pasadena, CA 91101

Corresponding author: Katia J. Bruxvoort, PhD; Department of Epidemiology, University of Alabama at Birmingham; 1665 University Blvd, Birmingham, AL, 35294; Email: kbruxvoort@uab.edu


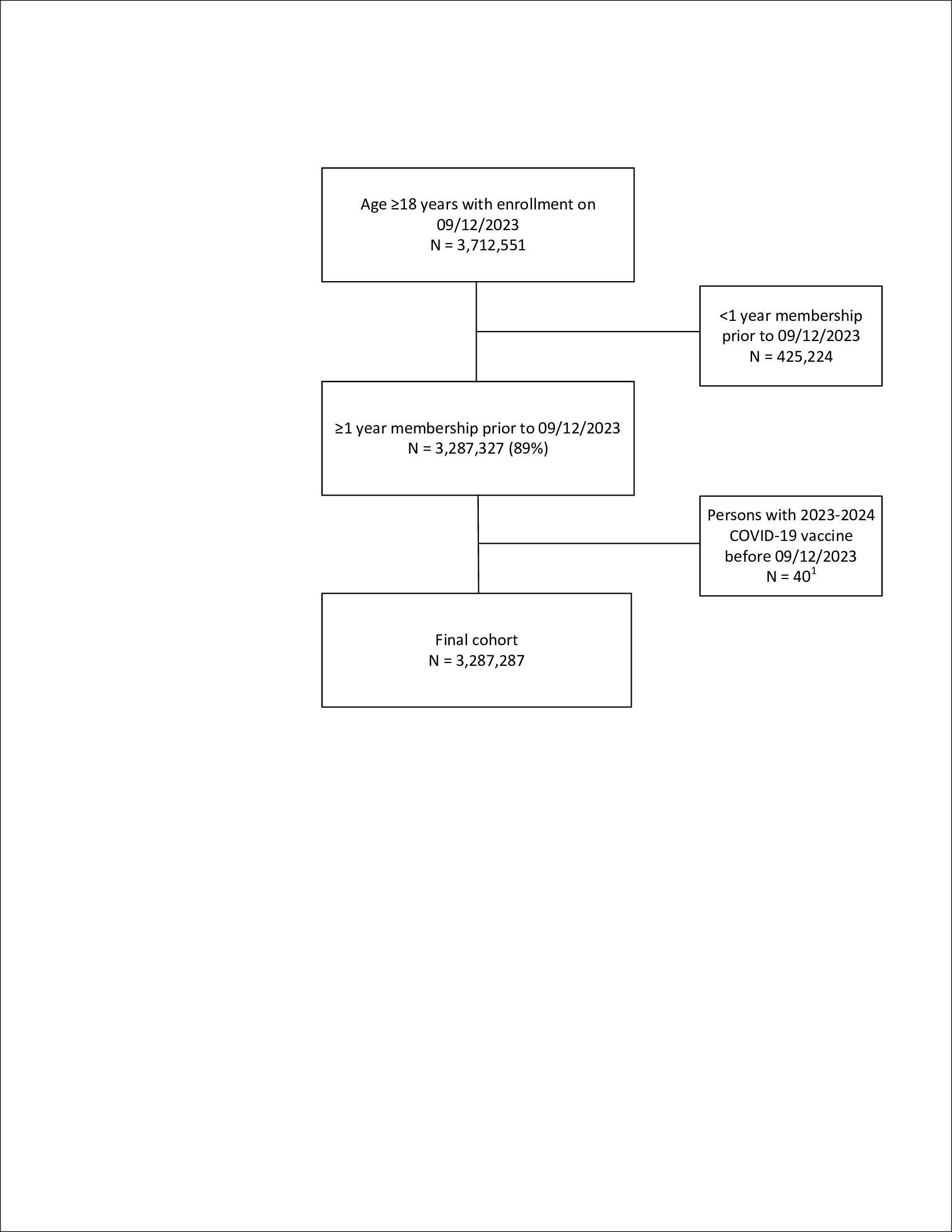
**Supplementary Figure 1: Flow chart for study cohort**

^1^ 40 individuals were excluded due to EHR data indicating that they received updated COVID-19 vaccine prior to September 12, 2023, before ACIP recommendations. These may have reflected errors in entry of vaccination records.

**Supplementary Table 1: Factors associated with not receiving updated COVID-19 vaccine in the post-hoc 2-variable model**

|  | **2-variable model** | |
| --- | --- | --- |
|  | **aOR** | **95% CI** |
| **Received 2022-2023 influenza vaccine** |  |  |
| Yes | ref |  |
| No | 4.65 | (4.60, 4.71) |
| **Received bivalent COVID-19 vaccine** |  |  |
| Yes | ref |  |
| No | 9.27 | (9.18, 9.36) |

Abbreviations: aOR, adjusted odds ratio; CI, confidence interval

**Supplementary Table 2: Performance of the post-hoc 2-variable model for not receiving updated COVID-19 vaccine**

|  | **2-variable model** | |
| --- | --- | --- |
| **Performance Measure** | **Development** | **Validation** |
|  | (n=1,643,644) | (n=1,643,643) |
| **Overall** |  |  |
| Brier | 0.110 | 0.110 |
| Brier scaled | 32.1% | 32.1% |
| R^2^ | 26.3% | 26.2% |
| **Discrimination** |  |  |
| C statistic (95 % CI) | 0.848 (0.85, 0.85) | 0.848 (0.85, 0.85) |
| Discrimination slope | 0.323 | 0.323 |
| **Calibration** |  |  |
| Calibration slope | 1 | 1 |

Abbreviations: CI, confidence interval
